# The Impact of the High-Fructose Corn Syrup on Cardiac Damage via SIRT1/PGC1-α Pathway: Potential Ameliorative Effect of Selenium

**DOI:** 10.1007/s12011-024-04081-z

**Published:** 2024-02-02

**Authors:** İlter İlhan, Halil Ascı, Halil İbrahim Buyukbayram, Orhan Berk Imeci, Mehmet Abdulkadir Sevuk, Zeki Erol, Fatih Aksoy, Adem Milletsever

**Affiliations:** 1 Faculty of Medicine, Department of Biochemistry, Suleyman Demirel University, Isparta, Turkey; 2 Faculty of Medicine, Department of Pharmacology, Suleyman Demirel University, Isparta, Turkey; 3 Faculty of Veterinary, Department of Food Hygiene and Technology, Burdur Mehmet Akif Ersoy University, Burdur, Turkey; 4 Faculty of Medicine, Department of Cardiology, Suleyman Demirel University, Isparta, Turkey; 5 Faculty of Veterinary, Department of Pathology, Burdur Mehmet Akif Ersoy University, Burdur, Turkey

**Keywords:** Selenium, HFCS, Mitochondrial biogenesis, SIRT1, PGC1-α

## Abstract

High-fructose corn syrup (HFCS) has been a subject of intense debate due to its association with cardiovascular risks. This study investigates the potential protective effects of selenium (Se) supplementation against cardiac damage induced by HFCS. Thirty-two male Wistar albino rats were divided into four equal groups: control, CS (20%-HFCS), CS with Se (20%-HFCS, 0.3 mg/kg-Se), and Se (0.3 mg/kg-Se) only. After a 6-week period, heart and aorta tissues were collected for histopathological, immunohistochemical, biochemical, and genetic analyses. HFCS consumption led to severe cardiac pathologies, increased oxidative stress, and altered gene expressions associated with inflammation, apoptosis, and antioxidant defenses. In the CS group, pronounced oxidative stress within the cardiac tissue was concomitant with elevated Bcl-2-associated X protein (Bax) expression and diminished expressions of B-cell-lymphoma-2 (Bcl-2), nuclear factor erythroid 2-related factor 2 (Nrf2), peroxisome proliferator-activated receptor gamma coactivator 1-alpha (PGC1-α), and silenced information regulator 1 (SIRT1). Se supplementation mitigated these effects, showing protective properties. Immunohistochemical analysis supported these findings, demonstrating decreased expressions of caspase-3, tumor necrosis factor-alpha (TNF-α), IL-1β, and vascular endothelial growth factor (VEGF) in the CS + Se group compared to the CS group. The study suggests that Se supplementation exerts anti-inflammatory, antioxidant, and antiapoptotic effects, potentially attenuating HFCS-induced cardiovascular toxicity. These findings highlight the importance of dietary considerations and selenium supplementation in mitigating cardiovascular risks associated with HFCS consumption.

**Supplementary Information:**

The online version contains supplementary material available at 10.1007/s12011-024-04081-z.

## Introduction

High-fructose corn syrup (HFCS) has become a subject of much debate in recent years. Many consumers and health experts express concerns about the potential dangers of HFCS. HFCS is a sweetener produced by an enzymatic hydrolysis process of corn starch. In this process, corn starch is typically derived from genetically modified corn. The resultant syrup, which includes both glucose and fructose, is widely used as a sweetener in a variety of processed foods and beverages. Due to genetic modification and its high fructose content, HFCS has been shown to increase the risk of significant diseases such as atherosclerosis, insulin resistance, obesity, and metabolic syndrome. These pathological conditions particularly have a negative impact on the cardiovascular system [1–3].

There is an ongoing debate about the potential effects of HFCS on increasing inflammation levels. Particularly with long-term exposure, it has been reported that myocytes, endothelial cells lining the blood vessels, and inflammatory cytokines such as interleukin-1 beta (IL-1β) and tumor necrosis factor-alpha (TNF-α) are elevated [4, 5]. One of the main reasons for this is believed to be the increased cellular oxidative stress [6].

Oxidative stress occurs when there is an excessive production of reactive molecules called free radicals or when the antioxidant defense mechanisms are insufficient in the body. The high fructose content in corn syrup is considered a factor that can increase oxidative stress in cells [7]. Free radicals generated during fructose metabolism can cause cellular damage and trigger the inflammatory process. Therefore, intracellular molecules that are affected by antioxidant substances, which can be used to reduce oxidative cell damage, are of great importance [8].

Silent information regulator 1 (SIRT1) plays a central role in protective intracellular mechanisms that respond to damage [9]. In addition to its antioxidant properties, SIRT1 also stands out for its antiapoptotic characteristics [10]. It activates nuclear factor erythroid 2-related factor 2 (Nrf2), which increases the production of protective enzymes such as glutathione peroxidase (GPx) and superoxide dismutase (SOD) through the antioxidant response element [11, 12]. Furthermore, through the deacetylation of peroxisome proliferator-activated receptor gamma coactivator 1-alpha (PGC1-α), it enhances mitochondrial biogenesis and exhibits antiapoptotic effects by reducing the Bax/Bcl-2 ratio [13]. Recent studies have demonstrated the protective properties of several agents that target key molecules like SIRT1 to exhibit these effects [14, 15].

Selenium (Se) is a crucial element in the composition of antioxidant enzymes like GPx and thioredoxin reductase, enhancing antioxidant activity and contributing to the safeguarding of cells and tissues from oxidative stress-induced harm. Studies have furnished evidence of Se anti-inflammatory and antioxidant properties in the context of cardiovascular health. Furthermore, Se has been demonstrated to elevate SIRT1 levels alongside antioxidant enzyme levels [16, 17].

This study aims to explore and elucidate the potential protective effects of Se supplementation against cardiac damage associated with the widespread use of HFCS. The global ubiquity of HFCS usage raises concerns about its detrimental impact on cardiac health. By investigating the potential protective properties of Se supplementation, we seek to furnish compelling evidence and a deeper understanding of the molecular mechanisms underlying protection against HFCS-induced cardiac damage.

## Materials and Methods

### Ethical Approval and Animal Care

Each of the 4 groups in our study consisted of 10 male Wistar albino rats (250–350 g), and the experiment lasted 6 weeks. Animal experiments received ethical approval from the local committee at Suleyman Demirel University (approval no:15.09.2022/ 06–74) and were conducted in compliance with the relevant European Communities Council Directive (86/609/EEC), adhering to recommended guidelines for animal care and experimentation.

### Experimental Design

Control group (*n* = 10): No active substance or drug was added to the drinking water for 6 weeks.

High-fructose corn syrup (CS) group (*n* = 10): A 55% fructose solution was mixed into the drinking water at a concentration of 20% for a period of 6 weeks [18, 19].

High-fructose corn syrup and selenium (CS + Se) group (*n* = 10): A 0.3 mg/kg dosage of Se was combined with a 55% fructose solution, which was then mixed into the drinking water at a concentration of 20% for 6 weeks [20].

Selenium (Se) group (*n* = 10): A 0.3 mg/kg dosage of selenium was mixed into the drinking water and administered for 6 weeks.

After the 6-week period, the rats were euthanized using ketamine (90 mg/kg) or xylazine (8–10 mg/kg) anesthesia. Half of the heart and aorta tissues collected following euthanasia were preserved in formaldehyde for subsequent histopathological analysis, while the remaining tissues were stored at − 80 °C for further biochemical and genetic examination.

### Histopathological Examination

During necropsy, heart and aorta samples were extracted and preserved in a 10% neutral formalin solution. Following a 48-h fixation period, the tissue samples underwent standard processing using a fully automated tissue processing system (Leica Microsystem, Germany) and were subsequently embedded in paraffin. After refrigeration, 5-µm sections were obtained using an automated rotary microtome (Leica microtome, Germany). Following overnight air-drying at room temperature, the sections underwent a series of alcohol and xylene treatments, were stained with hematoxylin–eosin, and were examined via a microscope.

### Immunohistochemical Examination

Paraffin blocks yielded four sets of sections, which were affixed to poly-L-lysine-coated slides and subjected to immunohistochemical staining for caspase-3 (cas-3) (anti-caspase-3 antibody (E-8): sc-7272), IL-1β (IL-1β (11E5):sc-52012), TNF-α (anti-TNFα antibody (52B83):sc-52746), and vascular endothelial growth factor (VEGF) (VEGF (JH1212):sc-57496). Immunostaining was performed using the streptavidin–biotin method following manufacturer instructions. Primary antibodies (Santa Cruz, USA) were used at a 1/100 dilution, and sections were incubated for 60 min. Biotinylated secondary antibody and streptavidin–alkaline phosphatase conjugate were employed for immunohistochemistry. The EXPOSE HRP/DAB Detection IHC kit (ab80436) from Abcam (UK) served as the secondary antibody, with diaminobenzidine (DAB) as the chromogen. Negative controls utilized antigen dilution solution in place of the primary antibody.

Immunohistochemical analysis involved separate examinations for each antibody. A semi-quantitative analysis, graded from (0) to (3), assessed the severity of cellular immunohistochemical reactions as follows: (0) = negative, (1) = focal weak staining, (2) = diffuse weak staining, and (3) = diffuse strong staining [21]. Evaluation encompassed 10 different areas under × 40 objective magnification in each section. Morphometric analyses and microphotography utilized the Database Manual CellSens Life Science Imaging Software (Olympus, Japan).

### Biochemical Analyzes

Rat heart tissues (approximately 150 mg each) were homogenized using the Ultra Turrax homogenizer (IKA® Werke, Germany) in a 1:9 (w/v) phosphate-buffered saline solution (pH: 7.4). Following homogenization, samples were centrifuged at 10,000 rpm for 10 min to determine oxidative stress. Total oxidant status (TOS), total antioxidant status (TAS), and oxidative stress index (OSI) levels in homogenized tissue samples were evaluated with an automated analyzer using Erel’s colorimetric method (Beckman Coulter, USA) [22, 23]. Then, the OSI value was determined by calculating OSI = [(TOS, µmol/l)/(TAS, mmol Trolox eq/l) × 100] [24]. The measurement of GPx activity was conducted following the Paglia and Valentine method, utilizing a commercially available kit from Randox Laboratories, UK. The SOD activity in heart tissue supernatants was assessed using the xanthine oxidase method with the utilization of the Ransod commercial kit, provided by Randox Laboratories, UK. Protein concentrations were measured using the Beckman Coulter AU5800 autoanalyzer (Beckman Coulter, USA). The outcomes were quantified in units per milligram of protein.

### RT qPCR Analyzes

RNA isolation from the homogenized tissues was carried out using the GeneAll RiboEx (TM) RNA Isolation Kit (GeneAll Biotechnology, Korea) following the manufacturer’s instructions. To assess the quantity and purity of the collected RNAs, the BioSpec-nano nanodrop device (Japan) was employed. For cDNA synthesis, 1 µg of RNA was utilized, and this process was performed with the A.B.T. ™ cDNA Synthesis Kit (Atlas Biotechnology, Turkey) in accordance with the provided protocol, using a thermal cycler. The primer sequences were designed based using the NCBI website. Table 1 details the primer sequences used in the investigation. On a Biorad CFX96 real-time PCR apparatus located in California, USA, gene expression levels were measured using the A.B.T.™ SYBR Master Mix (Atlas Biotechnology, Turkey). In this investigation, the GAPDH gene was utilized as the reference gene for normalization. The reaction mixture was made in accordance with the manufacturer’s instructions to yield a final volume of 20 µl. Following that, the mixture was put into a real-time qPCR equipment, and thermal cycling conditions were set according to the kit manual. Each sample underwent three replications. The RT-qPCR conditions included an initial denaturation step at 95 °C for 300 s, followed by denaturation at 95 °C for 15 s and annealing/extension at 56 °C for 30 s for a total of 40 cycles. After normalizing the data, the relative mRNA levels were computed using the 2-ΔΔCt formula [25].

**Table 1:** Primary sequences, product size, and accession numbers of genes

Genes	Primary sequence	Product size	Accession number
GAPDH (housekeeping)	F: AGTGCCAGCCTCGTCTCATA	248 bp	NM_017008.4
	R: GATGGTGATGGGTTTCCCGT		
Bcl-2	F: GGTGAACTGGGGGAGGATTG	102 bp	NM_016993.2
	R: AGAGCGATGTTGTCCACCAG		
Bax	F: AGGGTGGCTGGGAAGGC	93 bp	XM_039087751.1
	R: TGAGCGAGGCGGTGAGG		
Nrf2	F: GCCTTCCTCTGCTGCCATTAGTC	126 bp	NM_001399173.1
	R: TCATTGAACTCCACCGTGCCTTC		
PGC1-α	F: CGCACAACTCAGCAAGTCCTC	263 bp	XM_039092494.1
	R: CCTTGCTGGCCTCCAAAGTCTC		
SIRT1	F: GGTAGTTCCTCGGTGTCCT	152 bp	NM_001414959.1
	R: ACCCAATAACAATGAGGAGGTC		

*F* forward, *R* reverse, *GAPDH* glyceraldehyde-3-phosphate dehydrogenase, *Bcl2* B-cell lymphoma 2, *Bax* Bcl-2 associated X, *Nrf2* nuclear factor erythroid 2-related factor 2, *PGC1α* peroxisome proliferator-activated receptor gamma coactivator 1, *SIRT1* sirtuin 1.

### Statistical Analysis

Histopathological and genetic scores and tissue oxidative stress marker levels were compared between the groups with the one-way ANOVA and post hoc LSD or Duncan tests via using SPSS-15 package program. The level of statistical significance was considered as *p* < 0.05.

## Results

### Histopathological Examination

**Fig. 1:**
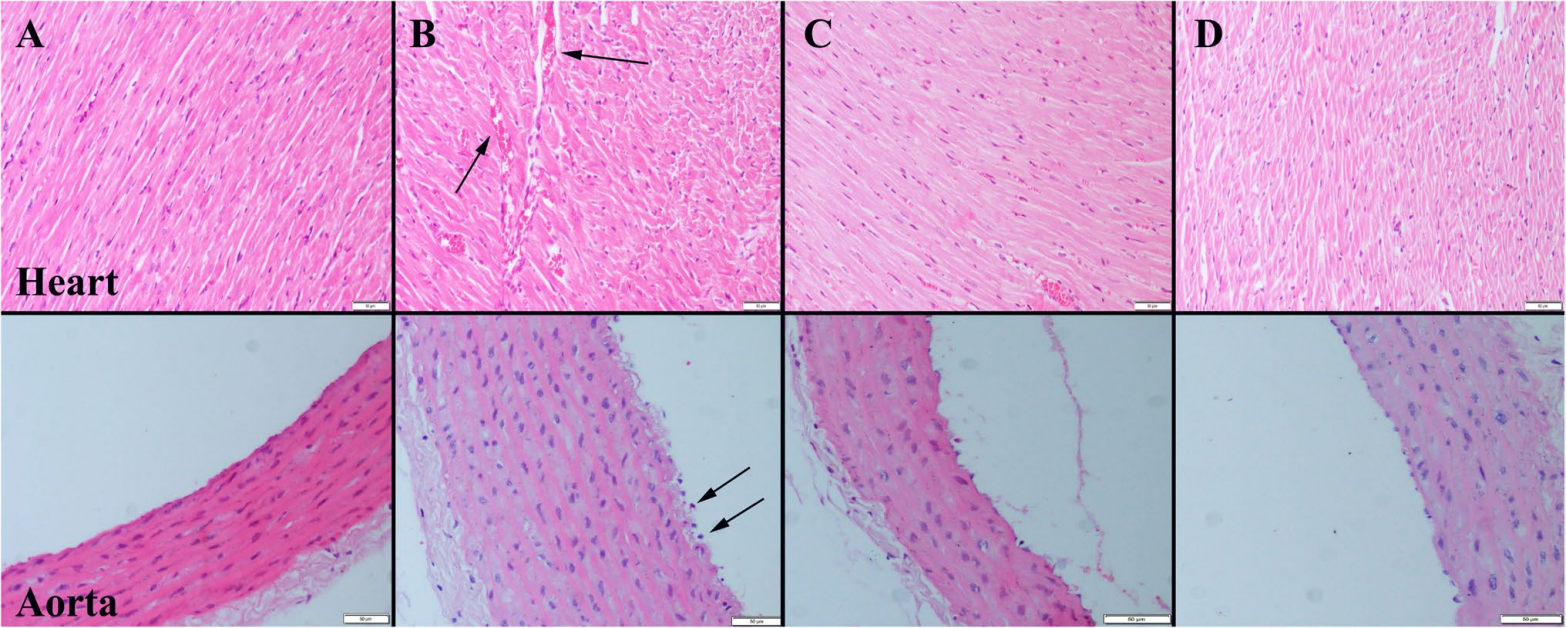
Representative histopathological figures between the groups. **A** Normal tissue histology in the Control group. **B** Marked hyperemia (arrows) in myocardial vessels and endothelial desquamations (arrows) in aortas. **C** Decreased hyperemia in CS + Se group. **D** Normal tissue architecture in Se group, HE, scale bars = 50 µm

Histopathological examination revealed the normal tissue histoarchitecture in Control and Se groups. At the CS group, severe hyperemia and edema were noticed in hearts. Furthermore, there was a noticeable decrease in endothelial cell count within this group. Se treatment alleviated pathological observations in both heart and aorta samples (Fig. 1).

### Immunohistochemical Examination

**Fig. 2:**
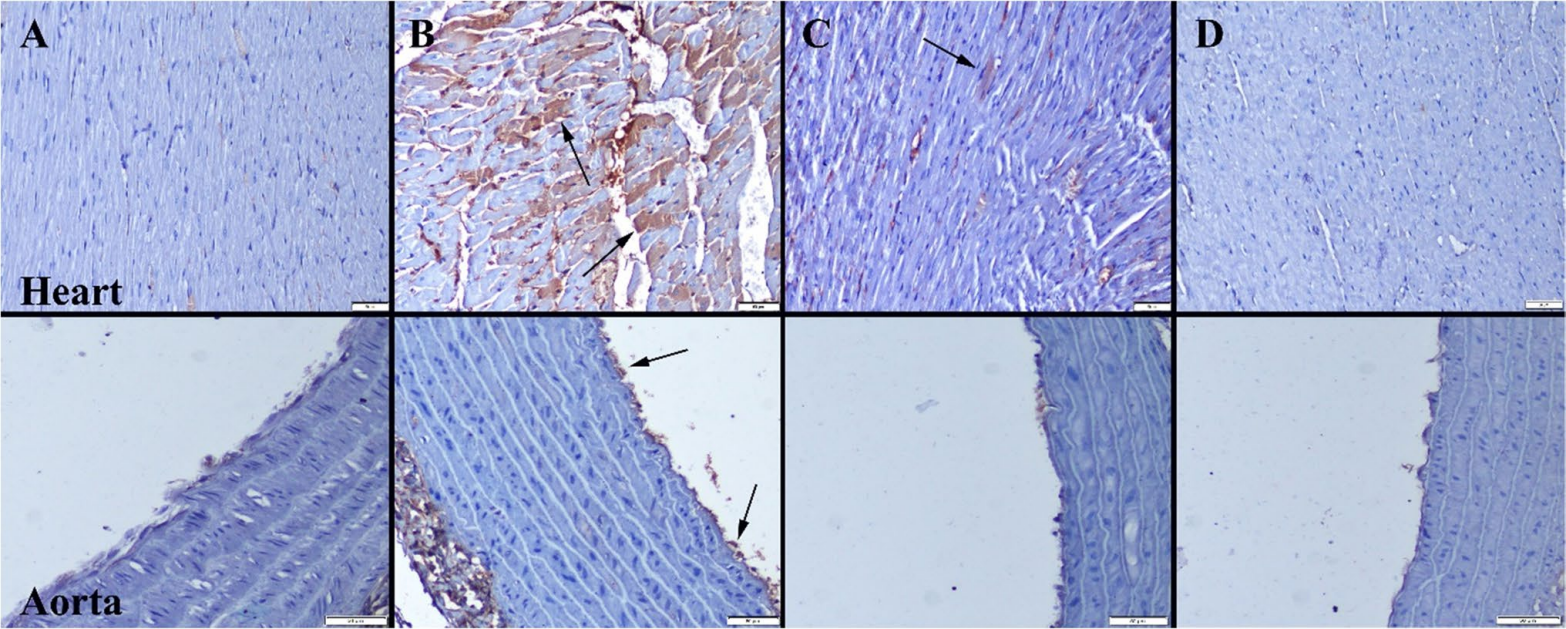
Cas-3 immunohistochemical findings of the heart (upper row) and aortas (below row) among the groups. **A** No expression in Control group. **B** Increased expression in myocardial and endothelial cells (arrows) in CS group. **C** Decreased expression in myocardial cells (arrow) in CS + Se group. **D** No expression in Se group. Streptavidin biotin peroxidase method, scale bars = 50 µm

**Fig. 3:**
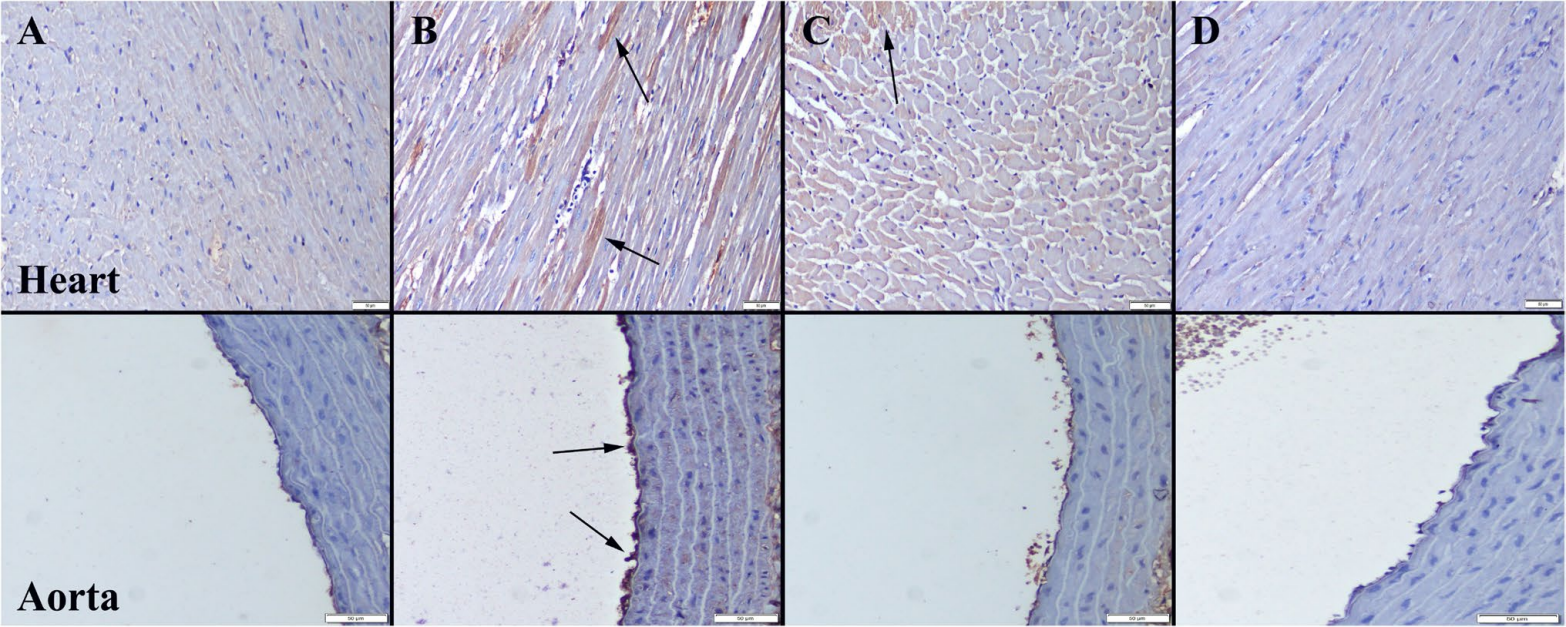
TNF-α immunoexpression findings of the heart (upper row) and aortas (below row) among the groups. **A** No expression in Control group. **B** Marked increase in expression in myocardial cells (arrows) in CS group. **C** Decreased expression in myocardial and endothelial cells (arrow) in CS + Se group. **D** No expression in Se group. Streptavidin biotin peroxidase method, scale bars = 50 µm

**Fig. 4:**
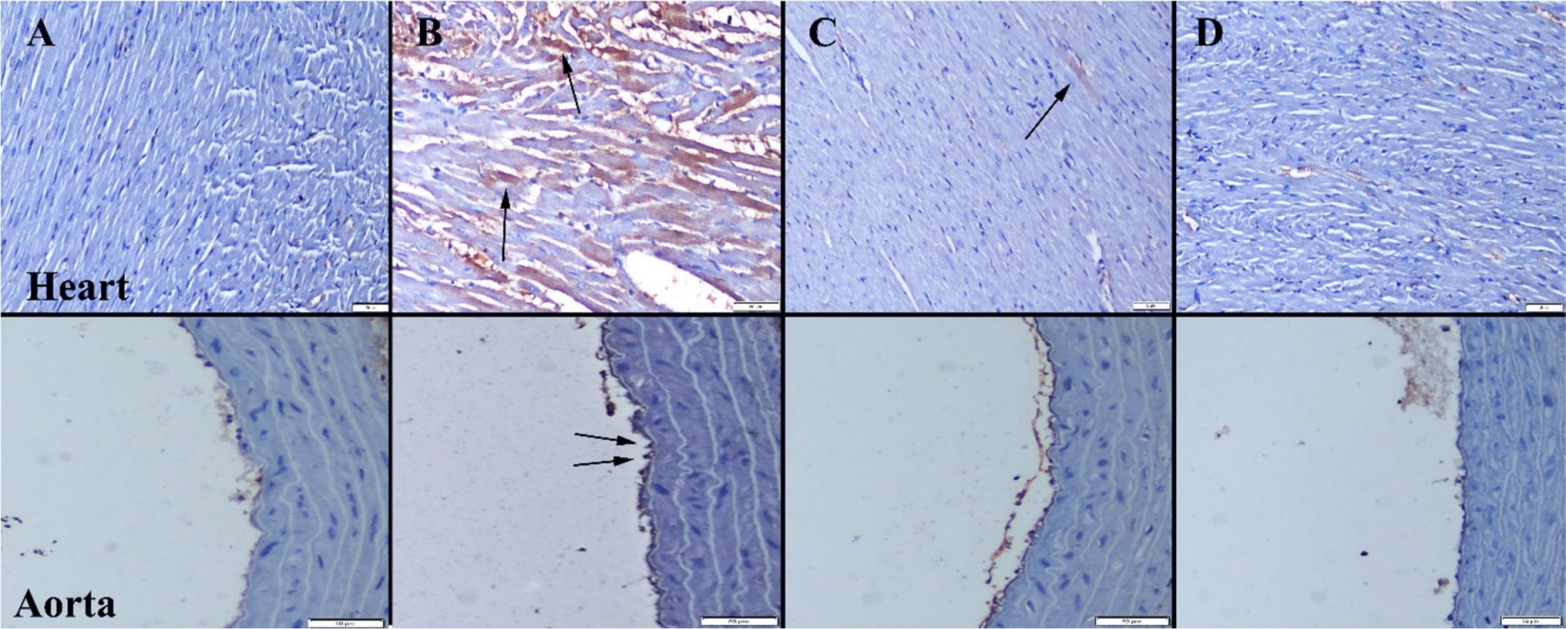
IL-1β immunoreactions of the heart (upper row) and aortas (below row) among the groups. **A** No expression in Control group. **B** Marked increase in expression in myocardial cells (arrows) in CS group. **C** Decreased expression in myocardial and endothelial cells (arrow) in CS + Se group. **D** Negative expression in Se group. Streptavidin biotin peroxidase method, scale bars = 50 µm

**Fig. 5:**
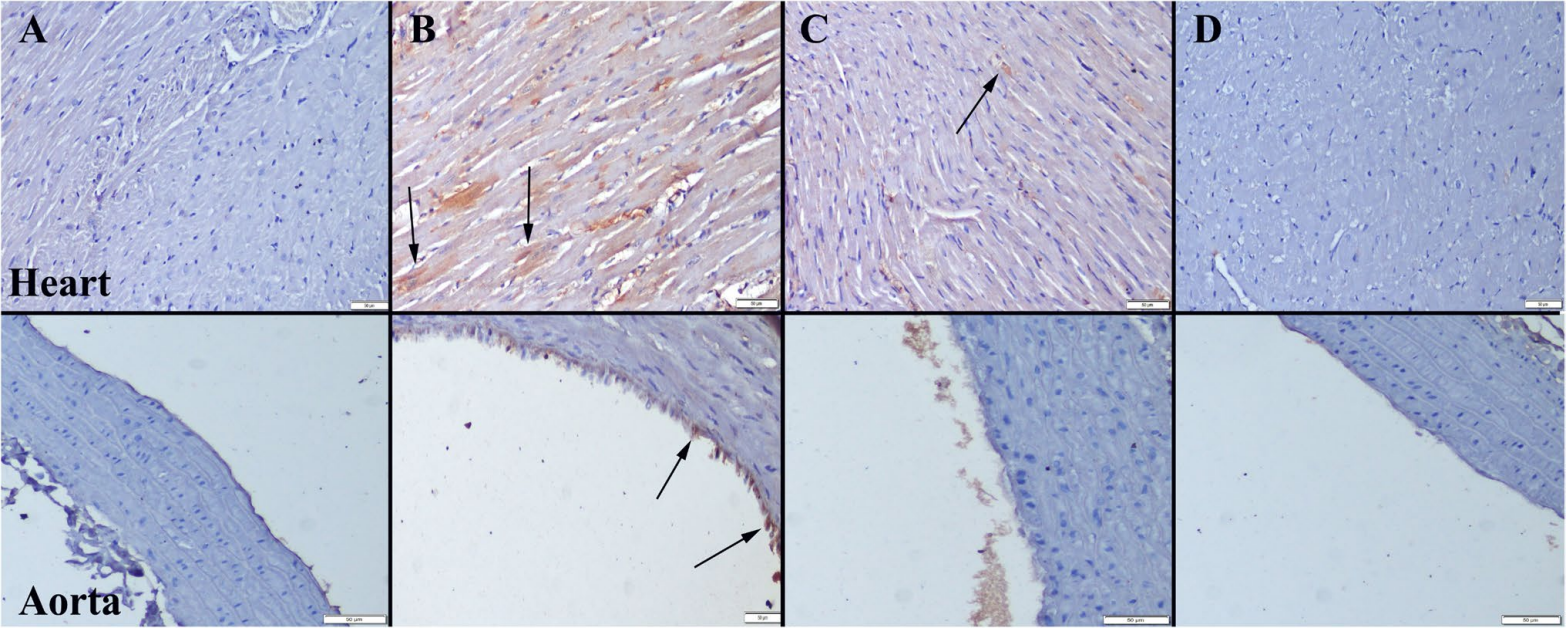
VEGF expressions of the heart (upper row) and aortas (below row) among the groups. **A** Expression not seen in Control group. **B** Increased expression in both myocardial and endothelial cells (arrows) in the CS group. **C** Decreased expression (arrow) in CS + Se group. **D** No expression in Se group. Streptavidin biotin peroxidase method, scale bars = 50 µm

At the immunohistochemical evaluation, increased expressions of cas-3, TNF-α, IL-1β, and VEGF were observed in the CS group. Se treatment caused a decrease in the expressions of all of the markers. Expressions were predominantly observed in myocardial cells within hearts and endothelial cells of aortas (Figs. 2, 3, 4, and 5). Statistical analysis results of immunohistochemical scores are shown in Table 2.

**Table 2:** Statistical analysis results of immunohistochemical between the groups

	Control	CS	CS + Se	Se	*p* value
Cas-3 heart	0.12 ± 0.12^a^	1.62 ± 0.51^b^	0.50 ± 0.18^a^	0.12 ± 0.12^a^	< 0.001
Cas-3 aorta	0.12 ± 0.12^a^	1.50 ± 0.53^b^	0.37 ± 0.18^a^	0.25 ± 0.16^a^	< 0.001
TNF-α heart	0.25 ± 0.16^a^	1.50 ± 0.18^b^	0.50 ± 0.18^c^	0.25 ± 0.16^a^	< 0.001
TNF-α aorta	0.12 ± 0.12^a^	1.25 ± 0.70^b^	0.25 ± 0.16^a^	0.25 ± 0.16^a^	< 0.001
IL-1β heart	0.25 ± 0.16^a^	1.50 ± 0.53^b^	0.37 ± 0.18^a^	0.25 ± 0.16^a^	< 0.001
IL-1β aorta	0.12 ± 0.12^a^	1.37 ± 0.74^b^	0.62 ± 0.26^a^	0.37 ± 0.26^a^	< 0.001
VEGF heart	0.37 ± 0.18^a^	1.50 ± 0.53^b^	0.87 ± 0.83^a^	0.25 ± 0.16^a^	< 0.001
VEGF aorta	0.12 ± 0.12^a^	2.12 ± 0.35^b^	0.62 ± 0.51^a^	0.25 ± 0.16^a^	< 0.001

Data expressed mean ± standard deviation (SD). Post hoc Duncan test following one-way ANOVA. Different letters in the same row represent significant difference at *p* < 0.001 level

These study findings indicated that CS caused pathological findings in the heart and aorta and Se has an ameliorative effect on CS-induced damage.

### Biochemical Analysis Results

**Fig. 6:**
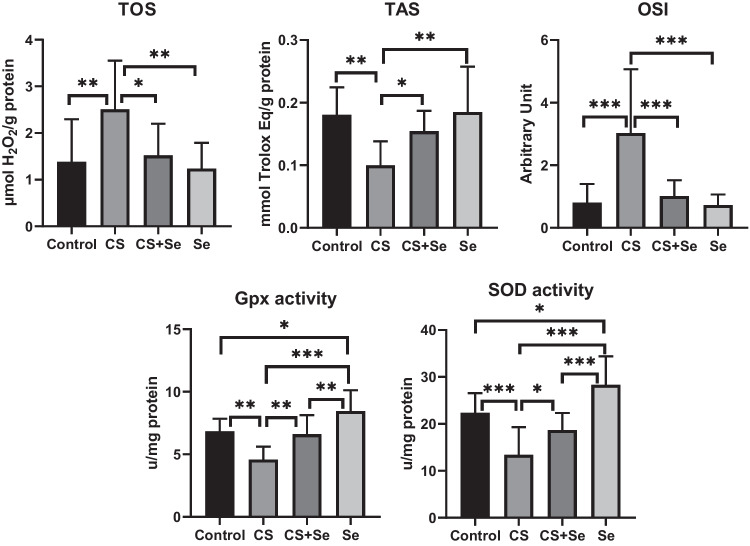
Oxidative stress parameters of heart tissue. Values are represented as means ± SD. Comparison between groups and results of oxidative stress markers were assessed by a one-way ANOVA test followed by post hoc LSD multiple comparison test. CS, corn syrup; Se, selenium; TOS, total oxidant status; TAS, total antioxidant status; OSI, oxidative stress index; GPx, glutathione peroxidase; SOD, superoxide dismutase. “***” represents *p* < 0.001, “**” represents *p* < 0.01, and “*” represents *p* < 0.05

The effect of CS on oxidative stress was evaluated with TOS, TAS, and OSI parameters in heart-homogenized tissue samples. TOS and OSI values were significantly elevated in the CS group than in the control group (*p* = 0.010 and *p* < 0.001; respectively). In contrast, TAS values were significantly lower in the CS group according to control (*p* = 0.003). When we compared the group given CS with the CS + Se and Se groups, we found that the TOS values decreased (*p* = 0.022 and *p* = 0.004, respectively), and the TAS values increased significantly in the CS + Se and Se groups (*p* = 0.036 and *p* = 0.002, respectively). Additionally, OSI was found to be higher in the CS group than in the CS + SE and SE groups (*p* < 0.001 for both groups) (Fig. 6).

In order to demonstrate the antioxidant effect of Se, the levels of GPx and SOD enzymes in the heart tissue were examined. In the CS group, both GPx and SOD enzyme activities were significantly diminished compared to the control group (*p* = 0.002 and *p* < 0.001, respectively). In the CS + Se group, significant elevation was observed in GPx and SOD enzyme levels compared to the CS group (*p* = 0.005 and *p* = 0.047, respectively), while only the group treated with selenium exhibited significantly higher GPx and SOD enzyme levels than all other groups (Fig. 6).

**Fig. 7:**
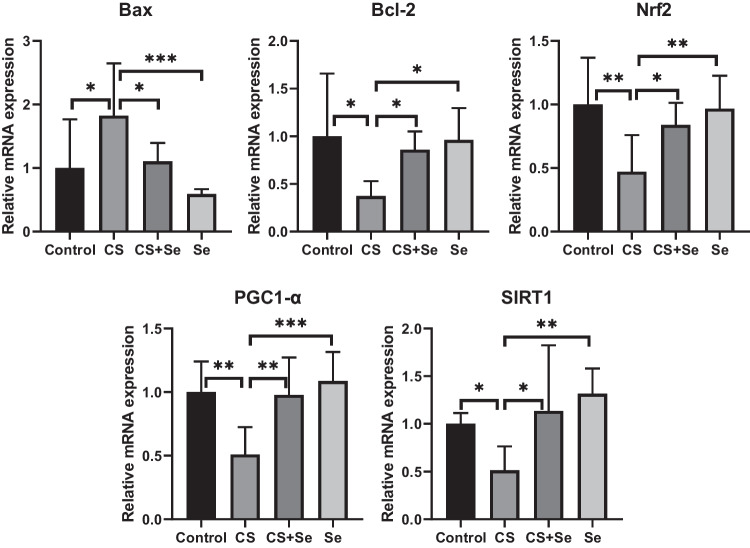
Relative mRNA expressions of Bax, Bcl-2, Nrf2, PGC1-α, and SIRT1 in cardiac tissue. Statistical analysis of mRNA relative fold change Ct values of genes was performed with one-way ANOVA and post hoc LSD test. CS, corn syrup; Se, selenium; Bax, Bcl-2-associated X protein; Bcl-2, B-cell lymphoma 2; Nrf2, nuclear factor erythroid 2–related factor 2; PGC1-α, peroxisome proliferator-activated receptor-gamma coactivator-1 alpha; SIRT1, sirtuin 1. “*” represents *p* < 0.05, “**” represents *p* < 0.01, and “***” represents *p* < 0.001

In the molecular examination of intracellular pathways related to apoptosis, oxidative stress, and inflammation, mRNA expressions of SIRT1, Nrf-2, Bax, Bcl-2, and PGC1-α were analyzed. In the evaluation of the CS group, a notable reduction in both SIRT1 and Nrf2 expressions was observed compared to the control group (*p* = 0.044 and *p* = 0.004, respectively). It was observed that Se supplementation in the treatment group increased the diminished expressions of SIRT1 and Nrf2 caused by the damage (*p* = 0.013 and *p* = 0.035, respectively). When comparing the CS group and the group receiving only Se, both SIRT1 and Nrf2 expressions were found to be higher in the Se group (*p* = 0.002 and *p* = 0.006, respectively). Although SIRT1 levels were increased in the Se group compared to the control group, it did not reach statistical significance. In the CS group, an increase in Bax expression and a decrease in Bcl-2 and PGC1-α expressions were observed compared to the control group (*p* = 0.023, *p* = 0.011, *p* = 0.003; respectively). These changes observed in the CS group were reversed with the Se treatment (*p* = 0.045, *p* = 0.042, and *p* = 0.004, respectively). When comparing the CS and Se groups, it was observed that the Se group had significantly lower Bax expression and higher Bcl-2 and PGC1-α expressions (*p* = 0.001, *p* = 0.016, *p* < 0.001, respectively) (Fig. 7).

## Discussion

This study indicated that HFCS consumption increased cas-3, TNF-α, IL-1β, VEGF, and Bax expressions and decreased Bcl-2, Nrf2, PGC1-α, and SIRT1 expressions. HFCS consumption also caused oxidative stress in the heart tissues of the rats. Se supplementation reversed these adverse effects of high fructose consumption.

Cardiovascular diseases (CVD) encompass a range of conditions affecting the heart tissue and blood vessels, representing the leading global cause of mortality. Mounting evidence suggests that increased fructose consumption elevates the risk of CVD, contributing to the development of hypertension, dyslipidemia, inflammation, and coronary heart disease [26]. Excessive consumption of fructose-sweetened beverages causes an increase in the risk of CVD. Even though raised CVD risk may be partially linked to fructose-related obesity or insulin-resistant conditions, direct fructose toxicity to the cardiovascular system is also possible [27]. Enhanced fructose intake stimulates the process of lipogenesis and leads to lipid accumulation within adipose tissue, consequently triggering an upsurge in the secretion of adipokines which cause systemic inflammation [28]. In addition, the elevation in fructose catabolism may stimulate the production of reactive oxygen species (ROS) through increased lipid peroxidation [29]. Also, HFCS intake leads to increased hepatic synthesis of uric acid. The escalated uric acid levels contribute to the onset of endothelial dysfunction by triggering oxidative stress and impairing endothelial nitric oxide production [30]. Moreover, it is observed that endothelial dysfunction induced by uric acid was linked to mitochondrial dysfunction [31]. Furthermore, the mitochondrial mass decreases in myocytes in the heart of fructose-fed rats [32]. Therefore, agents that support mitochondrial integrity may reduce harmful effects.

The primary hypothesis of this study posited that selenium supplementation enhances the expressions of SIRT1 and PGC1-α in the cardiac tissue. SIRT1 and PGC1-α are pivotal regulators involved in the facilitation of mitochondrial biogenesis, oxidative phosphorylation, expression of antioxidant enzymes, cellular migration, proliferation, and the inhibition of apoptosis. SIRT1, belonging to the sirtuin protein family, holds a pivotal position in metabolic pathways. It exerts its influence by deacetylating numerous target proteins, including histones in muscle, adipose tissue, heart, and endothelium. SIRT1 has demonstrated efficacy in ameliorating several degenerative conditions associated with neurodegeneration, cancer, and metabolic disorders, including glucose intolerance and insulin resistance [33]. Additionally, SIRT1 and PGC1-α control genes and proteins implicated in metabolism and sustain the proper functioning of mitochondria and peroxisomes, which are the essential sources of oxidative agents, especially in the heart (26). SIRT1 activates mitochondrial biogenesis and peroxisomal balance via the deacetylation of PGC1-α (25). SIRT1 and PGC1-α also stimulate the activity of antioxidant enzymes and decrease oxidative stress, and they can directly interact with nuclear factor-kappa B to decrease proinflammatory signaling in the heart and vasculature (27–29). The reduction in SIRT-1 expression concurrent with elevated oxidative stress represents an early hallmark of inflammation, foreshadowing subsequent cardiac dysfunction induced by a high-fructose diet [34]. In the present study, the CS group showed decreased Bcl2, Nrf2, PGC1-α, and SIRT1 expressions and increased Bax expression compared to the other groups. Accordingly, Se supplementation reversed this negative effect of HFCS. Savran et al. discovered that melatonin, which elevates SIRT1 levels, mitigated cardiac damage induced by oxidative stress and inflammation triggered by HFCS [35]. It has been shown in the literature that Se similarly increases SIRT1 levels in heart tissue [36]. Increased SIRT1 levels by Se acted in various ways. First, it influenced antioxidant enzyme synthesis and declined oxidative stress via GPx and SOD. GPx, a crucial selenoprotein essential for antioxidant activity, presents distinct subtypes in various tissues, including GPx 1, 3, and 4, specifically expressed in the heart [37]. Our previous study indicated that enhancing GPx activity could play a vital role in maintaining cardiovascular health in case of damage [21]. Beyond scavenging oxidant molecules, the increased expression of GPx4, in particular, has the potential to augment SOD enzyme activity [38]. There is also a positive correlation between SIRT1 and SOD levels, which is important for maintaining antioxidant balance [39]. Furthermore, Nrf2 stands as a pivotal factor in sustaining redox equilibrium, detecting oxidants, and orchestrating antioxidant defense mechanisms [40]. Additionally, Nrf2 overexpression exerts negative modulation on the NF-κB signaling pathway through intracellular mechanisms, resulting in the mitigation of inflammatory responses [41]. Similarly, we found that elevated SIRT-1 expression caused Nrf2 downregulation that resulted in the reduction of cytokine synthesis as TNF-α, IL-1β, and VEGF and alleviated inflammation.

Our findings also indicate that the consumption of HFCS activates the mitochondrial apoptosis pathway, as evidenced by elevated expressions of caspase-3 and Bax, coupled with diminished expressions of Bcl-2. In line with that, Cheng et al. have similarly shown the activation of mitochondrial apoptotic pathways in the cardiac tissue of rats fed with fructose [42]. Furthermore, Se demonstrated potential efficacy in mitigating mitochondria-dependent cardiac apoptosis in rats exposed to HFCS by reversing Bax, Bcl-2, and cas-3 expressions in our study. Mohamed et al. suggested that Se limits diabetic cardiac complications in rats by attenuating the mitochondrial cell death pathway through the reduction of apoptotic signals [43].

Histopathological and immunohistochemical findings corroborate our genetic and biochemical analysis results. It was observed that fructose feeding had a detrimental impact on the structural integrity of the heart. In the CS group, hyperemia, edema, and decreased endothelial cell count were observed in the vessels of the myocardium, and treatment with Se had a significant ameliorative effect on these pathologies.

## Conclusion

In conclusion, Se has anti-inflammatory, antioxidant, and antiapoptotic effects on HFCS-induced cardiovascular toxicity by increasing the expression of SIRT1. This elevation triggered PGC1-α activation and, as a result, led to a decrease in the production of cytokine synthesis from the nucleus, repressing inflammation and apoptosis and improving antioxidant enzyme levels to reduce oxidative stress. In light of these findings, potential interventions should be considered, including limiting dietary intake of foods containing HFCS in people at risk of CVD or supplementing these foods with Se as a preventive agent. To advance our understanding, future research should delve into the detailed molecular effects of Se, explore different dosages, durations of use, and routes of administration, and extend the evaluation to other organs and tissues. Also, conducting clinical studies with diverse sample groups across various risk categories is essential for a comprehensive evaluation of Se’s protective effects against CVD.

## Supplementary Information

Below is the link to the electronic supplementary material.

Supplementary file1 (JPG 967 KB)

## Data Availability

The datasets supporting the conclusions of this article are included within the article, and the original data of this study are available from the corresponding author upon reasonable request.
