# Correction: The Impact of the High-Fructose Corn Syrup on Cardiac Damage via SIRT1/PGC1-α Pathway: Potential Ameliorative Effect of Selenium

**DOI:** 10.1007/s12011-024-04100-z

**Published:** 2024-02-12

**Authors:** İlter İlhan, Halil Ascı, Halil İbrahim Buyukbayram, Orhan Berk Imeci, Mehmet Abdulkadir Sevuk, Zeki Erol, Fatih Aksoy, Adem Milletsever

**Affiliations:** 1 Faculty of Medicine, Department of Biochemistry, Suleyman Demirel University, Isparta, Turkey; 2 Faculty of Medicine, Department of Pharmacology, Suleyman Demirel University, Isparta, Turkey; 3 Faculty of Veterinary, Department of Food Hygiene and Technology, Burdur Mehmet Akif Ersoy University, Burdur, Turkey; 4 Faculty of Medicine, Department of Cardiology, Suleyman Demirel University, Isparta, Turkey; 5 Faculty of Veterinary, Department of Pathology, Burdur Mehmet Akif Ersoy University, Burdur, Turkey

**Correction: Biological Trace Element Research**

10.1007/s12011-024-04081-z

The published version of this article unfortunately contained a mistake.

The data availability statement should read “The datasets supporting the conclusions of this article are included within the article, and the original data of this study are available from the corresponding author upon reasonable request “.

The original article has been corrected.

