# Supplementary figures and images for: The Impact of the High-Fructose Corn Syrup on Cardiac Damage via SIRT1/PGC1-α Pathway: Potential Ameliorative Effect of Selenium
Source: Biol Trace Elem Res. 2024 Feb 2;202(11):5166–76. doi: 10.1007/s12011-024-04081-z (PMC11442503; doi:10.1007/s12011-024-04081-z)

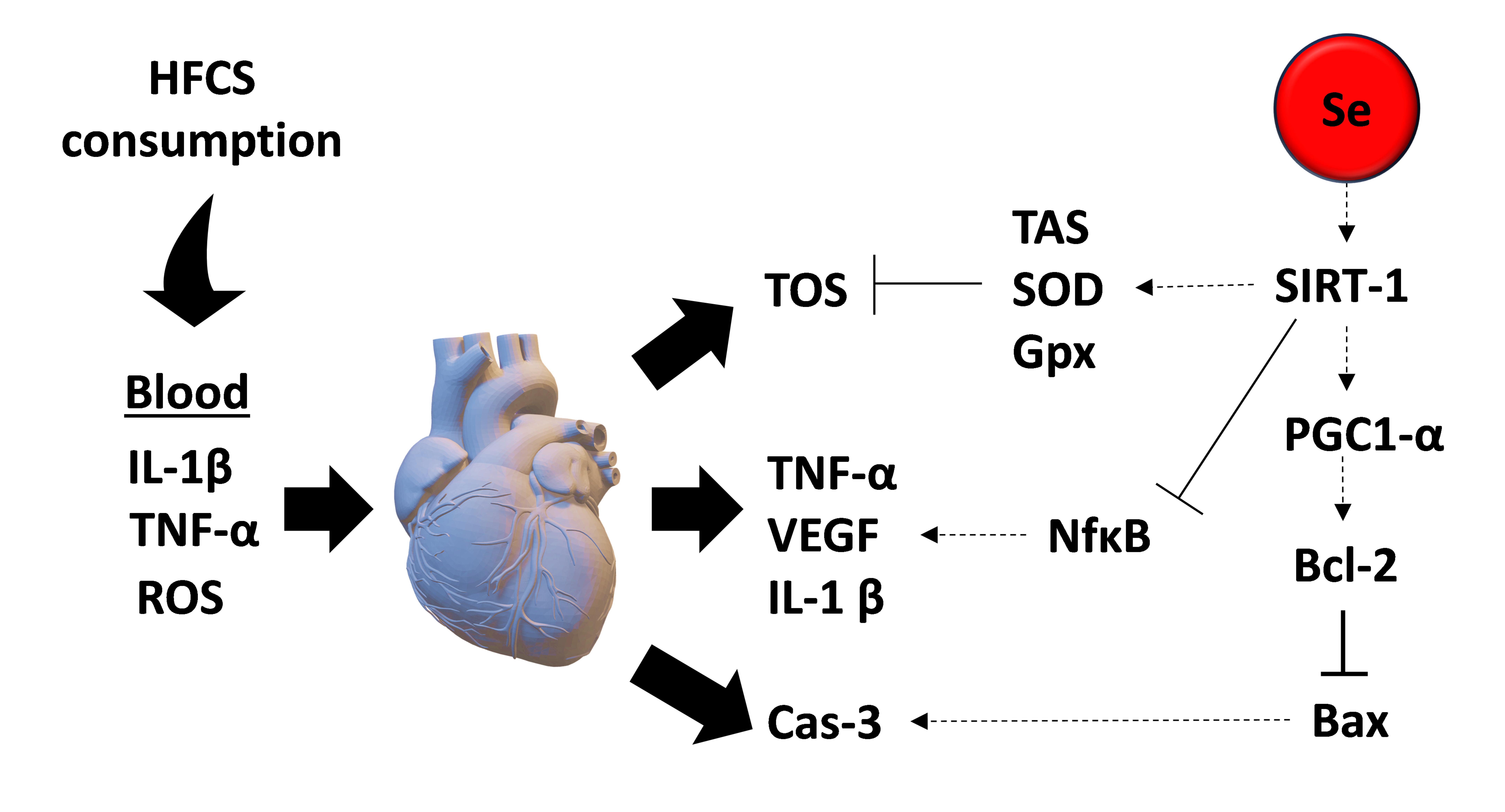

Supplement: Supplementary file 1 — Supplementary file1 (JPG 967 KB) [file 12011_2024_4081_MOESM1_ESM.jpg]
